# The genus *Coprinellus* (Basidiomycota; Agaricales) in Pakistan with the description of four new species

**DOI:** 10.3897/mycokeys.39.26743

**Published:** 2018-09-11

**Authors:** Shah Hussain, Muhammad Usman, Najam-ul-Sehar Afshan, Habib Ahmad, Junaid Khan, Abdul Nasir Khalid

**Affiliations:** 1 Centre for Plant Sciences and Biodiversity, University of Swat, Swat, Pakistan; 2 Department of Botany, University of the Punjab, Lahore 54590, Pakistan; 3 Centre for Undergraduate Studies, University of the Punjab, Lahore 54590, Pakistan; 4 Islamia College Peshawar, Pakistan

**Keywords:** *Coprinellus* section *Domestici*, *Coprinellus* sect. *Micacei*, coprinoid fungi, taxonomy

## Abstract

Mushrooms with a thin-fleshed pileus that becomes plicate on opening, deliquescent lamellae and dark brown to blackish basidiospores are commonly called coprinoid mushrooms. The genus *Coprinellus* is one of the important lineages of coprinoid mushroom in the family Psathyrellaceae. Species-level taxonomy in *Coprinellus* is based mainly on the presence or absence and the structure of veil and cystidia on the pileus, of cystidia on the lamellae and on basidiospore morphology. In this study, four new species of *Coprinellus* (*Co.campanulatus*, *Co.disseminatus-similis*, *Co.pakistanicus* and *Co.tenuis*) are described from Pakistan. Species descriptions are based on morphological and molecular data. Phylogenetic analyses based on nuc rDNA ITS region show that the new species *Co.campanulatus* and *Co.disseminatus-similis* are clustered in a clade including members of section Micacei; *Co.tenuis* falls in a clade with members of section Domestici; and *Co.pakistanicus* recovered in a separate clade adjacent to other recently described clades of genus *Coprinellus*. Morpho-anatomical descriptions of the new species and comparison with closely allied taxa are provided. With this study, the number of known species of *Coprinellus* in Pakistan has reached eight.

## Introduction

Coprinoid fungi form an important group of macrofungi and are striking in the field because of their deliquescent lamellae. Coprinoid mushrooms have generally a thin-fleshed pileus that becomes plicate on opening with deliquescent lamellae and dark brown to blackish basidiospores with germ-pore (Schafer 2010). The evolutionary lineages of coprinoid taxa are set amongst those that are not, or not fully coprinoid. Fully coprinoid genera include: *Coprinus* Pers. in Agaricaceae; *Coprinellus* P. Karst., *Coprinopsis* P. Karst. and *Parasola* Redhead, Vilgalys & Hopple in Psathyrellaceae. Certain species of *Leucocoprinus* Pat. (*L.birnbaumii*, *L.brebissonii*, *L.fragilissimus*) in Agaricaceae have a coprinoid combination of characters (Nagy 2011). Within the Bolbitiaceae, coprinoid taxa include: species of Conocybe Fayod belonging to section Candidae Watling, few *Bolbitius* Fr. species (*B.coprophilus*, *B.elegans*, *B.lacteus*, *B.reticulatus*, *B.subvolvatus*, *B.titubans*) and two species of *Galerella* Earle (*G.floriformis*, *G.nigeriensis*). Nevertheless, taken together, at least eight independent lineages with coprinoid fruiting bodies have hitherto been identified in the Psathyrellaceae (3), Bolbitiaceae (3) and Agaricaceae (2) (Matheny et al. 2006, Nagy 2011, Nagy et al. 2011, Tóth et al. 2013).

The genus *Coprinellus*, with approximately 80 described species, represents an independent lineage in Psathyrellaceae (Redhead et al. 2001, Walther et al. 2005, Vašutová et al. 2008, Padamsee et al. 2008, Nagy et al. 2011, 2012, 2013, Örstadius et al. 2015). These mushrooms are common saprotrophs of, for example, wood chip, leaf-litter and herbivore dung (Schafer 2010). Species of this genus are divided into three sections on the basis of veil anatomy and the presence or absence of cap pileocystidia. Section Domestici (Singer) D.J. Schaf. has a veil on the pileus in the form of floccose scales, consisting of chains of fusiform or subglobose cells, often with encrusted walls. In *Micacei* (Fr.) D.J. Schaf., veil remnants are present in the form of scattered, granulose flocks, often disappearing and consisting of globose cells arising from a matrix of narrow branched hyphae. In *Setulosi* (J.Lange) D.J. Schaf., the veil may be present or absent, but the pileus and stipe are covered with thin-walled pileocystidia and caulocystidia, respectively (Schafer 2010). However, Nagy et al. (2012) showed that these sections were not entirely consistent with the molecular phylogeny, in particular because clades corresponding to sections *Micacei* and *Domestici* each included some setulose species.

Previously, only 18 species of coprinoid mushrooms have been reported from Pakistan (Ahmad 1980, Hussain et al. 2016, 2017, 2018). These include two species of *Coprinus* (*C.comatus* (O.F. Müll.) Pers., *C.hookeri* Berk.); four of *Coprinellus* (*Co.disseminatus* (Pers.) J.E. Lange, *Co.marculentus* (Britzelm.) Redhead, Vilgalys & Moncalvo, *Co.micaceus* (Bull.) Vilgalys, Hopple & Jacq. Johnson, *Co.radians* (Desm.) Vilgalys, Hopple & Jacq. Johnson); five of *Coprinopsis* (*Cop.atramentaria* (Bull.) Redhead, Vilgalys & Moncalvo, *Cop.jonesii* (Peck) Redhead, Vilgalys & Moncalvo, *Cop.lagopus* (Fr.) Redhead, Vilgalys & Moncalvo, *Cop.macropus* (Berk. & Broome) Redhead, Vilgalys & Moncalvo, *Cop.patouillardii* (Quél.) G. Moreno); and seven of *Parasola* (*P.auricoma* (Pat.) Redhead, Vilgalys & Hopple, *P.glabra* Hussain, Afshan, Ahmad & Khalid, *P.lilatincta* (Bender & Uljé) Redhead, Vilgalys & Hopple, *P.malakandensis* Hussain, Afshan & Ahmad, *P.plicatilis* (Curtis) Redhead, Vilgalys & Hopple, *P.pseudolactea* Sadiqullah, Hussain & Khalid, *P.setulosa* (Berk. & Broome) Redhead, Vilgalys & Hopple).

During explorations of basidiomycetous fungi in Pakistan in 2014–2017, some interesting collections of *Coprinellus* were encountered. Upon further examination, it was discovered that these collections represent four new species. The current report provides species descriptions based on morphological characters and molecular phylogenetic analyses of nuc rDNA internal transcribed spacers (ITS1-5.8S-ITS2 = ITS). With this study, the number of known species in *Coprinellus* in Pakistan increases to eight.

## Materials and methods

### Sampling and morphology

Samples were collected in August–September 2014–2017, in the Malakand district of Khyber Pakhtunkhwa and Pabbi district of Punjab, Pakistan. Specimens were photographed, tagged and morphological features including size, shape and colour of basidiomata were noted. For colour designations, the Munsell (1975) colour system was followed. For anatomical study, slides were prepared in 5% aqueous KOH (w/v). Anatomical features, including size and shape of basidiospores, basidia, cheilocystidia, pileipellis and position of germ-pore in basidiospores, were studied using a light microscope (MX4300H, Meiji Techo Co., Ltd., Japan). Data of morpho-anatomical features were recorded from at least 20 measurements. In case of basidiospores, at least 50 spores were measured in face view and side view at a magnification of 1000× and measurements were rounded to the nearest 0.5 µm. Basidiospore measurements are presented as: length range × breadth range × width range. Q values were calculated as: Q_1_ = length divided by breadth; Q_2_ = length divided by width (Nagy et al. 2010). Specimens studied during this work are deposited in the Herbarium of University of the Punjab, Lahore (LAH) and the Herbarium of University of Swat, Swat, Pakistan (SWAT).

### DNA extraction, PCR amplification and sequencing

For DNA extraction, we used the DNeasy Plant Mini Kit (Qiagen, Redwood City, California, USA). We amplified nuc rDNA internal transcribed spacer region (ITS) using the primer combination ITS1F/ITS4 (White et al. 1990). The polymerase chain reaction (PCR) was performed in a 25 µl reaction volume: containing 2.5 µl 10× Econo Taq Buffer (Lucigen, Middleton, Wisconsin, USA), 0.5 µl dNTPs, 1.25 µl of each primer (10 µM/µl), 0.125 µl of Econo Taq® DNA Polymerase (Lucigen), 14.375 µl H_2_O and 5 µl DNA template. PCR amplification were performed with 4 min initial denaturation at 95°C, followed by 34 cycles of 50 s at 94°C, 40 s at 54°C, 50 s at 72°C and a final extension of 7 min at 72°C followed the last cycle. The PCR products were purified using a QIAquick PCR purification kit (Qiagen Inc., Valencia, California, USA). Sequencing was performed using a Bigdye terminator cycle sequencing kit (Applied Biosystems, Foster City, California, USA). Sequencing reactions were purified using Pellet Paint (Novagen, Madison, Wisconsin, USA) and were run on an Applied Biosystems 377 XL automated DNA sequencer. Sequence chromatograms were compiled with Sequencher 4.1 software (GeneCodes Corporation, Ann Arbor, Michigan, USA). Sequences generated for this study are deposited in GenBank (MH366735–MH366737, MH753663–MH753670).

### Alignment and phylogenetic analyses

Consensus sequences were generated from both forward and reverse primer reads in BioEdit sequence alignment editor version 7.2.5 (Hall 1999) and then homology searches were performed at the National Center for Biotechnology Information (NCBI) Web site using BLAST. These BLAST results, along with the sequences recently employed in the phylogeny of *Coprinellus* (Nagy et al. 2012), were used in the phylogenetic analyses. DNA sequences were aligned in Clustal X 2.1 (Larkin et al. 2007). *Psathyrellacandolleana* (Fr.) Maire was used as outgroup. Sequence alignment was deposited in TreeBase (http://purl.org/phylo/treebase/phylows/study/TB2:S23199).

Phylogenetic inference was conducted using Bayesian and Maximum Likelihood (ML) methods. For Bayesian inference, we used BEAST 1.6.2 (Drummond and Rambaut 2007) with a Markov chain Monte Carlo (MCMC) coalescent approach. For tree prior, a Yule-type speciation model (Gernhard 2008) was used in all simulations and the starting tree was randomly generated. Four independent runs were undertaken. Chain length was 20 million generations, with a sampling frequency of 1000. Tracer 1.6 (Rambaut et al. 2014) was used to check the effective sample size (ESS) and burn-in values were adjusted to achieve an overall ESS of ≥200. A Maximum Clade Credibility Tree (MCCT) with 20% burn-in was generated using TreeAnnotator 1.6.2 (Drummond and Rambaut 2007). Maximum Likelihood analyses were run in RAXML-VI-HPC (Stamatakis 2006) under the GTRCAT model. Branch support was calculated by 1000 bootstrap replicates. Nodes were considered strongly supported when the maximum likelihood bootstrap (MLB) values were ≥ 70% and Bayesian posterior probability (BPP) values were ≥ 0.95.

## Results

### Phylogenetic analyses

The ITS dataset comprises 97 sequences and the resulting alignment was 708 bp in length. Phylogenetic trees reconstructed using both Bayesian and ML methods were mostly congruent with each other. Taxa of *Coprinellus* were recovered in seven clades (Figure 3). Clades I–IV consisted of species of section Setulosi, three corresponding to clades described in Nagy et al. (2012). Clade I, corresponding to core *Setulosi* clade, was recovered with strong statistical support (BPP/ML 1/98). Clade II corresponded to *Sabulicola* clade with a single species *Co.sabulicola* L. Nagy, Házi, Papp & Vágvölgyi with strong statistical support (1/100). Clade III was the new species *Coprinelluspakistanicus*, forming an independent lineage (1/100). Clade IV corresponded to *Eurysporoid* clade with strong support (1/100). Clade V consisted of species of the *Micacei* clade of Nagy et al. (2012), including *Co.disseminatus* (morphologically placed in section Setulosi) along with species of morphological section Micacei and recovered with strong statistical support (1/99). The two new species *Coprinelluscampanulatus* and *Co.disseminatus-similis* fall in this clade. *Coprinelluscampanulatus* formed a sister clade (weak statistical support) with *Co.micaceus* (Bull.) Vilgalys, Hopple & Jacq. Johnson and *Co.truncorum* (Scop.) Redhead, Vilgalys & Moncalvo and would be placed in morphological section Micacei. *Coprinellusdisseminatus-similis* (1/100) formed a sister clade with *Co.disseminatus* (Pers.) J.E. Lange, adding a further setulose species to this group. Clades VI and VII collectively consisted of species of the *Domestici* clade of Nagy et al. (2012), including species that would be placed morphologically in section Setulosi. The fourth new species, *Co.tenuis*, formed a sister clade (1/100) with *Co.curtus* (Kalchbr.) Vilgalys, Hopple & Jacq. Johnson.

### Taxonomy

#### Coprinellus campanulatus

Taxon classificationFungiAgaricalesPsathyrellaceae

Hussain & Ahmad sp. nov.

MB825477

Figures 1E and 4

##### Diagnosis.

The diagnostic features of *Coprinelluscampanulatus* are: campanulate pileus with greyish-olive tinge, surface with glistening clusters of micaceous veil at maturity, dark yellowish-brown centre, basidiospores 8.0–10.5 × 5.5–6.5 × 4.5–5.5 µm, spores mitriform in face view and cylindrical to amygdaliform in side view.

##### Type.

PAKISTAN: **Khyber Pakhtunkhwa**, Qaldara, Dargai, Malakand, 480 m alt., gregarious on wood chip, 14 Aug 2014, *S. Hussain*, SH144 (LAH-SH-144, holotype); GenBank accession ITS: MH753667.

##### Etymology.

The epithet “*campanulatus*” (Latin) refers to the campanulate shape of the pileus of this species.

##### Macroscopic characters.

Pileus at young stage 3–8 × 3–7 mm, ovoid to parabolic, light orange-yellow (7.5YR 9/8) to pale orange-yellow (7.5YR 9/4), surface pruinose; at mature stage 25–40 × 10–15 mm, pulvinate to campanulate, light greyish-olive (10Y 5/2) to greyish-olive (5Y 3/2), centre slightly campanulate, strong yellowish-brown (10YR 4/8) to dark yellowish-brown (10YR 1/2); surface finely furfuraceous to granulose, with clusters of micaceous-glistening veil, bright white, plicate from near centre to margin; context membranous to submembranous. Lamellae adnexed, narrow, with fimbriate edge, crowded with 1–4 series of lamellulae, pale orange-yellow (7.5YR 9/4) at young stage, dark yellowish-brown at maturity (10YR 2/2). Stipe 70–100 × 3–7 mm, equal, white, surface smooth, context hollow. Annulus absent with a membranous layer at the base. Odour pungent. Not tasted.

##### Microscopic characters.

Basidiospores (7.0–)8.0–10.5(–11.5) × (5.0–)5.5–6.5(–7.0) × (4.0–)4.5–5.5(–6.0) µm, on average 9.4 × 5.7 × 5.1 µm, Q_1_ = 1.6, Q_2_ = 1.8, av. Q = 1.7; in face view mitrifrom, triangular to ellipsoid; in side view cylindrical, amygdaliform to ellipsoid; dark brown to blackish in KOH, smooth, thick-walled, with truncate base, apiculus visible, germ-pore 1.5–2.5 µm wide, central, prominent, pale to hyaline. Basidia 19–29 × 7–10 µm, cylindrical, clavate to subclavate, hyaline, 4-spored. Cheilocystidia 36–47 × 35–45 µm, globose to subglobose, hyaline, abundant. Pleurocystidia absent. Pileipellis an epithelium of loosely arranged globose to subglobose or ellipsoid, hyaline to light olive, thin-walled elements, 30–80 × 25–60 µm. Veil composed of globose to subglobose cells, 50–90 µm diam., slightly thick-walled, yellowish-brown in KOH. Caulocystidia absent. Clamp connections rarely present.

##### Habitat and distribution.

Gregarious on woody litter under *Morusalba*, so far only known from lowland northern Pakistan.

##### Additional specimens examined.

PAKISTAN: Khyber Pakhtunkhwa, Malakand, Qaldara, on woody pasture, 14 August 2014, *S. Hussain*, SH144 (SWAT SHP144).

##### Comments.

The main distinguishing features of *Coprinelluscampanulatus* are: campanulate pileus with greyish-olive tinge, dark yellowish-brown centre, veil on pileus in the form of micaceous-glistening clusters which are composed of globose to subglobose cells and basidiospores 8.0–10.5 × 5.5–6.5 × 4.5–5.5 µm, spores mitriform in face view and cylindrical to amygdaliform in side view. Based on veil anatomy, *Co.campanulatus* belongs in sect. Micacei. *Coprinellusmicaceus* and *Co.truncorum* are most closely related to *Co.campanulatus* amongst the species sampled for our phylogenetic analyses. The new species *Co.campanulatus* with pulvinate to campanulate pileus can be differentiated from *Co.micaceus* and *Co.truncorum*, which have broadly convex pilei. At maturity, the pileus is light brown in *Co.micaceus* and *Co.truncorum* when compared to *Co.campanulatus* with greyish-olive pileus. On basis of spore morphology, *Co.campanulatus* can be differentiated from *Co.micaceus*. Basidiospores in *Co.micaceus* are slightly smaller (6.5–10.0 × 4.5–7 µm), lacrimiform to submitriform or mitriform in face view, conical towards base (Keirle et al. 2004, Uljé 2005). In *Co.micaceus*, voluminous, broadly clavate, (sub)globose to ellipsoid pleurocystidia up to 150 × 70 µm are present, in *Co.campanulatus* pleurocystidia are absent. Also, in *C.micaceus*, caulocystidia are abundant, in *Co.campanulatus* absent. Spores of *Co.truncorum* are 8.5–9.0 × 5.5–6 µm, ellipsoid in all views, not distinctly lentiform, with very broad central to slightly eccentric germ pore, broadly rounded apex, not truncate, smooth, dark grey to grey brown or black (Keirle et al. 2004, Uljé 2005).

**Figure 1.**
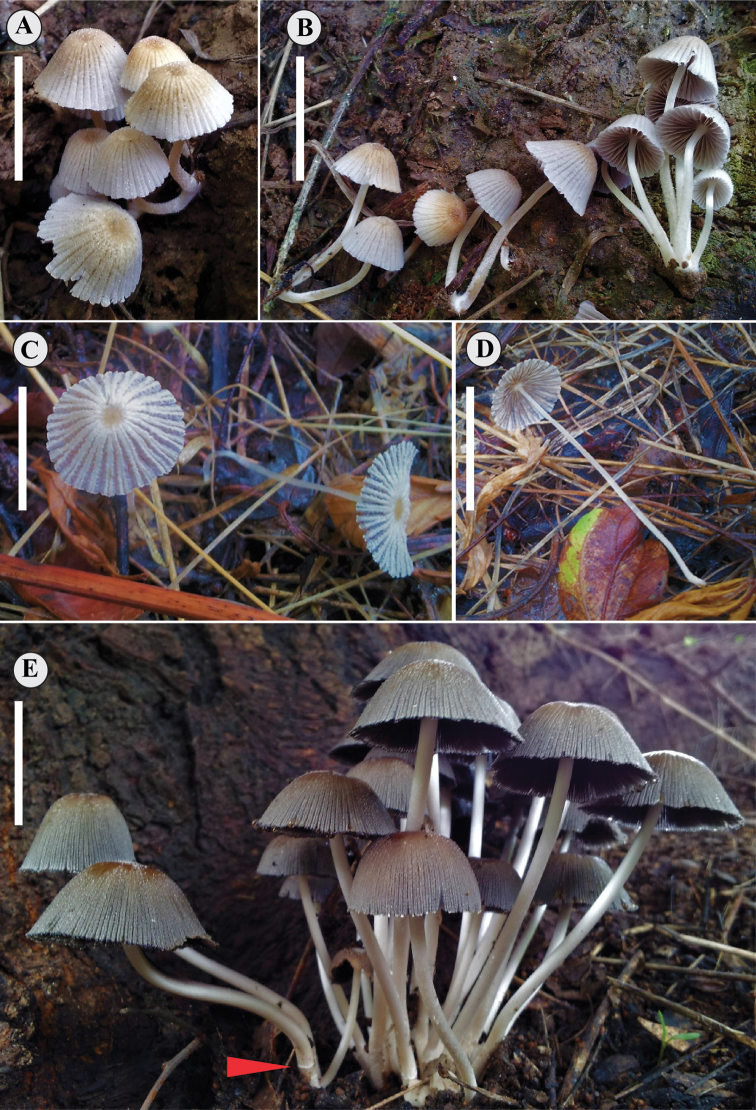
Basidiomata of species of *Coprinellus*. **A–B***Coprinellusdisseminates-similis* (holotype SHCr3W) **C–D***Coprinellustenuis* (holotype SHP10) **E***Coprinelluscampanulatus* (holotype SH144). The arrow shows remnants of membranous annulus. Scale bars: 20 mm.

#### Coprinellus disseminatus-similis

Taxon classificationFungiAgaricalesPsathyrellaceae

Hussain sp. nov.

MB825478

Figures 1A–B and 5

##### Diagnosis.

The most important features of *Co.disseminatus-similis* are: pileus parabolic to campanulate, greyish-brown, with umbonate centre; surface pruinose to pulverulent, with sparse micaceous-glistening veil, bright white, deeply plicate from centre to margin; basidiospores 8.0–9.0 × 5.0–5.5 × 4.5–5.5 µm, in face view ellipsoid to cylindrical or obovoid, in side view ellipsoid to amygdaliform, smooth, thick-walled, with truncate base, germ-pore central, 0.5–1.0 µm wide.

##### Type.

PAKISTAN: Khyber Pakhtunkhwa, Malakand, Sarogai, 450 m alt., gregarious on wood chips, 23 Sept 2014, *S. Hussain*, SHCr3w (SWAT-SHCr3w, holotype); GenBank accession ITS: MH753670.

##### Etymology.

“*Similis*” (Latin) meaning like, referring to the similarity of the new species to *Coprinellusdisseminatus*.

##### Macroscopic characters.

Pileus at young stage cylindrical and closed, 3–5 × 3–7 mm, whitish to light greyish (2.5Y 7/4), surface pruinose, slightly plicate toward margin; at mature stage 15−20 × 20 mm, parabolic to campanulate to umbonate, light greyish-brown (7.5YR 6/2) to greyish-yellowish-brown (7.5YR 6/2); with umbonate centre, in old specimens centre papillate, centre moderate orange (2.5YR 6/8) to brownish-orange (2.5YR 5/8); surface pruinose to pulverulent, with sparse micaceous-glistening veil, bright white, deeply plicate from centre to margin; context membranous. Lamellae sinuate to uncinate, distant with 0–2 lamellulae, initially white, fading with age and dark greyish-brown at maturity. Stipe 20−40 × 1 mm, equal, central, white, surface pruinose to pulverulent with sparse micaceous-glistening veil, context hollow, annulus absent. Odour pungent, not tasted.

##### Microscopic characters.

Basidiospores (7.5–)8.0–9.0(–9.5) × (4.5–)5.0–5.5(–6.0) × (4.0–)4.5–5.5(–6.0) µm, on average 8.5 × 5.2 × 4.9 µm, Q_1_ = 1.53–1.7, Q_2_ = 1.7–1.9, av. Q = 1.6; in face view, ellipsoid to cylindrical or obovoid, in side view, ellipsoid to amygdaliform, dark brown to blackish in KOH, smooth, thick-walled, with truncate base, germ-pore central, 0.5–1.0 µm wide. Basidia 26−30 × 7−10 µm, clavate to cylindrical, 2 to 4−spored, hyaline. Cheilocystidia 70−165 × 11−15 µm, cylindrical, narrowly clavate to narrowly utriform, some with subcapitate apex, abundant, smooth, hyaline. Pleurocystidia absent. Pileipellis a loosely arranged euhymeniderm with narrowly utriform to utriform pileocystidia, 118−165 × 23−28 µm, light-brownish to hyaline, smooth. Veil elements 20–40 µm, globose to subglobose, greyish-brown, smooth. Clamp connection not observed.

##### Habitat and distribution.

Gregarious on leaf litter under *Populusalba* and *Morusalba*, so far only known from lowland northern Pakistan.

##### Additional specimens examined.

PAKISTAN. Khyber Pakhtunkhwa: Malakand, Sarogai, on leaf litter under *Populusalba* and *Morusalba*, 22 Sept 2014, *S. Hussain*, SH-Cr3-b (SWAT SH-Cr3-b).

##### Comments.

The new species would be placed in sect. Setulosi because of its pileocystidia. However, as with *Co.disseminatus*, which it resembles and is close to in the molecular phylogram, *Co.disseminatus-similis* falls in a clade along with members of section Micacei that lack such pileocystidia, underlining the need to update the formal description of the sections. Both these species share basidiospore morphology. However, they differ on the basis of: (i) pileus shape and colour, (ii) cheilocystidia and (iii) pileocystidia and veil anatomy. In *Co.disseminatus*, initially the pileus is (sub)globose or ovoid, then hemispherical or obtusely conical to convex, rarely flat, the fruit bodies often form in very large groups and are initially very pale, almost white, darkening as the spores mature; cheilocystidia are absent along most of the gill edge; pileocystidia are lageniform with cylindrical neck and rounded, rarely subcapitate, apex and large 50–200 × 15–24 µm; and veil elements are globose to subglobose, generally with golden brown incrustations (Uljé and Bas 1991, Uljé 2005). In *Co.disseminatus-similis*, at young stage, the pileus is cylindrical and closed, parabolic to campanulate to umbonate at mature stage, with papillate centre in some old specimens; cheilocystidia are large (70−165 × 11−15 µm), narrowly clavate to narrowly utriform, some with subcapitate apex; pileocystidia are narrowly utriform to utriform; and veil elements are globose to subglobose and smooth. Using ML and Bayesian analyses, *Coprinellusverrucispermus* (Joss. & Enderle) Redhead, Vilgalys & Moncalvo is another species close to *Co.disseminatus-similis*. Spores in *Co.verrucispermus* are substantially larger (11.0–14.5 × 7.0–9.0 µm), ellipsoid to slightly amygdaliform, chestnut brown, apiculus slight, warty with perisporial sac and central germ pore (Uljé and Bas 1991, Keirle et al. 2004).

#### Coprinellus pakistanicus

Taxon classificationFungiAgaricalesPsathyrellaceae

Usman & Khalid sp. nov.

MB825483

Figures 2 and 6

##### Diagnosis.

The distinguishing features of *Coprinelluspakistanicus* are: light yellowish-green to greyish-yellow pileus, surface smooth with sub-membranous context, basidiospores 8.5–11.5 × 6.5–8.0 × 5.5–6.5 µm, on average 10 × 7.4 × 6.2 µm, in face view broadly ellipsoid, obovoid to phaseoliform, in side view ovoid, ellipsoid to obovoid, base not truncate, apiculus visible in side view, germ-pore central.

##### Type.

PAKISTAN: Punjab, Pabbi Forest Park, 286 m alt., 11 Aug 2016, *M. Usman* and *Abdul N. Khalid*, MU37 (Holotype LAH35323); GenBank accession ITS: MH366736.

##### Etymology.

The specific epithet “*pakistanicus*” refers to the holotype locality of this species.

##### Macroscopic characters.

Pileus 25–35 mm diam, convex to plan, with depressed centre, light yellow green (2.5GY 8/6) to greyish-greenish-yellow (7.5Y 7/4); surface smooth with sparsely pulverulent to granulose, deeply plicate from centre towards margin; centre depressed to slightly papillate, orange yellow (7.5YR 6/8); context sub-membranous, light greyish (10Y 5/2). Lamellae free, crowded, regular, dark brown to blackish, with 0–2 series of lamellulae. Stipe 27–50 × 1 mm, central, hollow, smooth, white, with slightly bulbous base. Annulus and volva absent. Odour and taste not recorded.

##### Microscopic characters.

Basidiospores (7–)8.5–11.5(–12) × (6.0–)6.5–8.0(–8.5) × (–5.0)5.5–6.5(–7.0) µm, on average 10 × 7.4 × 6.2 µm, Q_1_ = 1.4, Q_2_ = 1.6, av. Q = 1.3; in face view, broadly ellipsoid, obovoid to phaseoliform, in side view, ovoid, ellipsoid to obovoid, base not truncate, apiculus slightly visible, germ-pore central, smooth, slightly thin-walled, dark brown to blackish in KOH. Basidia 13.5–32 × 8.5–12 µm, clavate to narrowly clavate, hyaline, smooth, 2- to 4-spored, sterigmata up to 4 µm in length. Cheilocystidia 42–75 × 14–25 µm, cylindrical to lageniform, hyaline with crystals usually at the apex of cystidium. Pleurocystidia absent. Pileipellis irregular epithelium, 3.5–7.5 µm diam., pale to hyaline in KOH. Pileocystidia 30–90 × 9–24 µm, lageniform to cylindrical with tapering neck and obtuse apex, pale to hyaline in KOH. Veil rounded to globose cells, 15–25 µm diam., slightly thick-walled, yellowish in KOH. Clamp connection present.

##### Habitat and distribution.

Scattered on moist soil, under trees of *Acacianilotica* and *A.modesta*, so far only known from lowland northern Pakistan.

##### Additional specimens examined.

PAKISTAN. Punjab: Pabbi Forest Park, 286 m alt., 20 Aug 2016 & 2017, M. Usman, Abdul N. Khalid and A. Hameed, MU07, MU39 (LAH35324 and LAH35325).

##### Comments.

In phylogenetic analyses, *Coprinelluspakistanicus* forms Clade III, adjacent to the *Sabulicola* and *Eurysporoid* clades of Nagy et al. (2012) and morphologically would be placed in sect. Setulosi. The new species is compared with the following species of sect. Setulosi: *Co.bisporus* (J.E. Lange) Vilgalys, Hopple & Jacq. Johnson, *Co.cinereopallidus* L. Nagy, Házi, Papp & Vágvölgyi, *Co.congregatus* (Bull.) P. Karst., *Co.pellucidus* (P. Karst.) Redhead, Vilgalys & Moncalvo, *Co.radicellus* Házi, L. Nagy, Papp & Vágvölgyi and *Co.sabulicola* L. Nagy, Házi, Papp & Vágvölgyi.

In *Co.bisporus*, the pileus is small, up to 20 mm diam., ochre or pale brown; with dark red-brown basidiospores; cheilocysticdia subglobose, ovoid, ellipsoid to broadly utriform and smaller in size (24–40 × 16–23 µm) when compared to *Co.pakistanicus* (Prydiuk 2010). In *Co.cinereopallidus*, basidiospores are larger 12.1 × 6.5 µm, ellipsoid to subamygdaloid, not lentiform (Nagy et al. 2012). Similarly, *Co.congregatus* with pileus up to 20 mm in diam., cream-coloured, at centre ochre-brown to light brown, cheilocystidia subglobose, ovoid to ellipsoid, sometimes utriform, 22–50 × 15–36 µm in size (Prydiuk 2010). *Coprinelluspellucidus* with substantially small pileus (7 mm diam.), basidiospores 9.25 × 4.75 µm, elongate-ellipsoid to cylindrical-ellipsoid, with subglobose cheilocystidia, 20–25 × 14–22 µm (Prydiuk 2010). Pileus in *Co.radicellus* up to 10 mm diam., cream coloured to dark melleous-brown, expanding to convex applanate with uprolled margin, basidiospores on average 9.48 × 4.91 µm, reddish-brown, ellipsoid to subcylindrical, with globose to subglobose or clavate cheilocystidia, 9–20 × 8–14 µm in size (Házi et al. 2011). *Co.sabuilcola* has concave, warm reddish-brown pileus, basidiospores on average 17.3 × 10.9 µm, cheilocystidia 17–32 × 12.5–27 µm, globose to vesiculose or broadly ellipsoid (Nagy et al. 2012).

**Figure 2.**
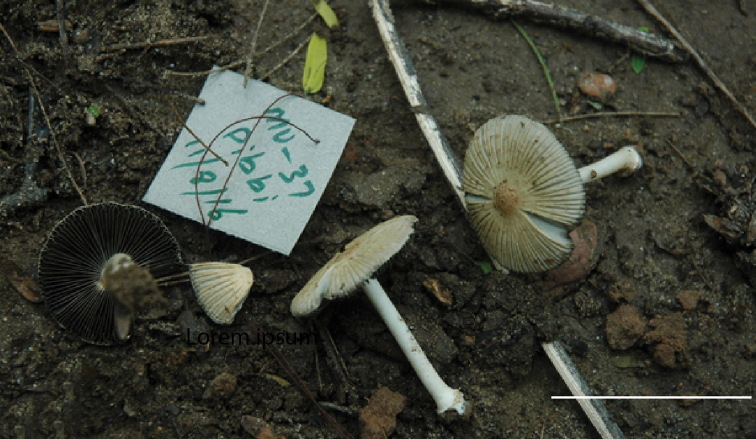
Basidiomata of *Coprinelluspakistanicus* Holotype (MU37). Scale bar: 20 mm.

#### Coprinellus tenuis

Taxon classificationFungiAgaricalesPsathyrellaceae

Hussain sp. nov.

MB825479

Figures 1C–D and 7

##### Diagnosis.

The new species *Coprinellustenuis* can be recognised by its thin and membranous pileus, surface glabrous and furred, deeply plicate towards margin; lamellae sinuate to uncinate; basidiospores 10.5–14.5 × 8.0–9.5 × 6.5–8.5 µm, in face view, broadly ellipsoid to ovoid, in side view, slightly pyriform to ellipsoid, usually with truncate base, apiculus mostly not visible, with eccentric germ-pore, 1.5–2 µm wide.

##### Type.

PAKISTAN: Khyber Pakhtunkhwa, Malakand, Qaldara, 430 m alt., solitary on leaf litter, 7 July 2014, *S. Hussain*, SHP10 (SWAT-SH-P10, holotype); GenBank accession ITS: MH753663.

##### Etymology.

“*tenuis*” (Latin) meaning thin, referring to the membranous pileus of the new species.

##### Macroscopic characters.

Pileus 15–20 mm diam, pulvinate to convex to plane, light greyish-brown (7.5YR 5/2) to light brown (5YR 6/4); surface glabrous, furred, deeply plicate from centre towards margin; centre truncately conical, moderate reddish-orange (10R 5/8) to greyish-reddish-orange (2.5YR 5/6); context membranous. Lamellae sinuate to uncinate, distant, with 0–2 series of lamelullae, light greyish-brown (7.5YR 5/2) to light brown (5YR 6/4), lamellae edge blackish and fimbriate to eroded. Stipe 40–60 × 1 mm, equal, cylindrical, surface scabrous, white, translucent, fragile, context hollow.

##### Microscopic characters.

Basidiospores (9.0–)10.5–14.5(–15.5) × (7.5–)8.0–9.5(–10.5) × (5.0–)6.5–8.5(–9.0) µm, on average 13.1 × 9.0 × 7.8 µm; Q_1_ = 1.25–1.49, Q_2_ = 1.57–1.63, av. Q = 1.45; in face view, broadly ellipsoid to ovoid, in side view, slightly pyriform to ellipsoid, usually with truncate base, apiculus mostly not visible, germ-pore eccentric, 1.5–2 µm wide, wall 1.5 µm thick, dark brown to almost black. Basidia 22–24 × 9–12 µm, clavate, 2- to 4-spored, hyaline in KOH. Cheilocystidia 22–30 × 19–28 µm, rounded to globose, abundant, hyaline. Pleurocystidia absent. Pileocystidia 78–94 × 10–12 µm, lageniform to cylindrical with rounded apex, elongated rod shape neck with rounded enlarged base, hyaline in KOH. Caulocystidia 50–67 × 9–11 µm, narrowly clavate to clavate, with rounded to obtuse apex, cylindrical base. Veil comprised of rounded to subglobose cells, arranged in short chain, thick-walled with encrusted walls, dark brown, with terminal cell 17–23 × 12–15 µm.

##### Habitat and distribution.

Scattered on leaf litter under *Acaciamodesta*, so far only known from lowland northern Pakistan.

##### Additional specimens examined.

PAKISTAN. Khyber Pakhtunkhwa: Malakand, Qaldara, on leaf litter under *Acaciamodesta*, 10 July 2014, *S. Hussain*, SH10 (SWAT SH-10).

##### Comments.

*Coprinellustenuis* with thin membranous pileus, shows similarities with *Co.curtus*. Both these species can be differentiated on (i) pileus morphology (ii) basidiospore shape and (iii) habitat. Pileus is deeply plicate in both these species, in *Co.tenuis* pileus is glabrous and furred; however, there is no furcation in the pileus of *Co.curtus*. Spores in *Co.curtus* are substantially smaller (8.0–10.0 × 5.5–7.0 µm), ellipsoid to ovoid in face view, narrowly ellipsoid or phaseoliform in side view, apiculus often not visible, with a distinct central to slightly eccentric germ-pore, not truncate. Basidiospores in *Co.tenuis* are larger (10.5–14.5 × 8.0–9.5 × 6.5–8.5 µm), in face view broadly ellipsoid to ovoid, in side view slightly pyriform to ellipsoid, usually with truncate base, apiculus mostly not visible, with eccentric germ-pore of 1.5–2 µm diam. *Coprinelluscurtus* has a substrate preference and is most commonly collected from herbivores’ dung as opposed to *Co.tenuis* basidioma on leaf litter (Uljé and Bas 1991).

## Discussion

The genus *Coprinellus* is one of the most species-rich genera in Psathyrellaceae, with approximately 80 described species (Kirk et al. 2008, Nagy et al. 2012, Gomes and Wartchow 2014). Species of *Coprinellus* have been classified in three sections, reflecting earlier sub-sections of *Coprinus* sensu lato, primarily based on veil anatomy and the presence or absence of cap pileocystidia (Schafer 2010). The most recent phylogenetic study of this genus by Nagy et al. (2012), does not provide evidence for the monophyly of morphologically based sections of previous classifications (Orton and Watling 1979, Uljé 2005, Schafer 2010).

**Figure 3.**
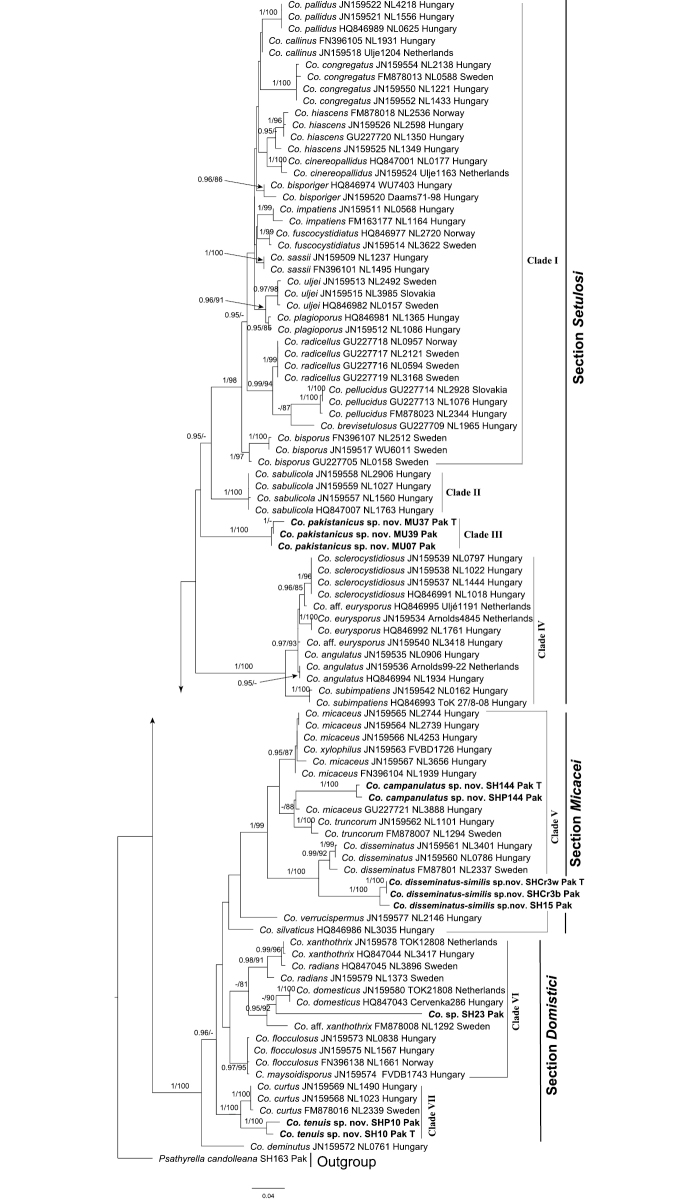
Phylogenetic inference of *Coprinellus* species inferred from 97 ITS sequences, with species names following GenBank accessions, specimen voucher numbers and country. Values above branch node represent Bayesian posterior probabilities (BPP) and maximum likelihood bootstrap (MLP), the new species are represented with bold fonts and T represents the holotype collection.

In the phylogeny we present here, based on ITS sequences, the genus is recovered in seven clades (Figure 3). In morphology-based taxonomy, species in section Setulosi have setules on their pilei and the majority of such species recovered as a non-monophyletic lineage consisting of four clades in this study. Clade I, corresponding to core *Setulosi* clade in the Nagy et al. (2012) phylogeny, is a large group of species with the characteristic setules on the pileus. Clade II corresponds to *Sabulicola* clade with a single species *Co.sabulicola* L. Nagy, Házi, Papp & Vágvölgyi. This species bears some unique features compared with other *Coprinellus* species; amongst these are relatively large basidiospores (15–22 × 10–13 µm), lack of a pedicel on the cystidia, habitat in dry, sandy sites and short, capitate pileocystidia with incrusted base (Nagy et al. 2012). Clade III represents the new species *Coprinelluspakistanicus*. This species has ellipsoid to phaseoliform basidiospores, cylindrical to lageniform cheilocystidia, pileocystidia lageniform to cylindrical with tapering neck and obtuse apex, veil with rounded to globose cells, slightly thick-walled, clamp connections present amongst most tissues. Clade IV, corresponding to the *Eurysporoid* clade (fig. 1 of Nagy et al. 2012), was inferred with strong statistical support (1/100) and consisted of some well-studied species, forming a basal group in this phylogeny. Amongst the species, there are *Coprinelluseurysporus* (M. Lange & A.H. Sm.) Redhead, Vilgalys & Moncalvo, *Co.sclerocystidiosus* (M. Lange & A.H. Sm.) Vilgalys, Hopple & Jacq. Johnson, *Co.subimpatiens* (M. Lange & A.H. Sm.) Redhead, Vilgalys & Moncalvo.

Clade V includes species of sect. Micacei, along with *Co.disseminatus* and our new species *Co.disseminatus-similis*, reflecting the *Micacei* clade of Nagy et al. 2012. It also includes *Co.verrucispermus* and *Co.deliquescens* (=*Co.silvaticus*), which were placed in the *Domestici* clade in that study, although data would allow a plausible phylogenetic position for those two species in the *Micacei* clade (Nagy et al. 2012, p.256). Taxa in section Micacei have a veil in the form of glistening mica-like granules, consisting of thin-walled globose cells in a matrix of narrow branched hyphae. The granules can be easily washed off by rain drops, causing difficulties in differentiation (Schafer 2010). Rich veil coverage on the pileus was suggested as a character linking the non-setulose and setulose species in both the *Domestici* and *Micacei* clades, the key feature for the *Micacei* clade being mitriform shaped basidiospores (Nagy et al. 2012).

Clade VI and VII, if taken together, would collectively correspond to the *Domestici* clade, inferred as a non-monophyletic group in *Coprinellus*. Species in clade VI have a veil consisting of floccose scales, made up of generally thick-walled, yellow-brown chains of inflated, ellipsoid or globose cells (thin-walled and hyaline in *Co.flocculosus*) and correspond to section Domestici. "*Coprinusmaysodisporus*" in Nagy et al. 2012 ("*Coprinusmaysoidisporus*" in GenBank) appears to refer to collection FVDB1743 and appears to relate to a collection of a provisionally named species "*Coprinusmaydisiformis*", close to *Co.xanthothrix*, from Washington State, USA in 1972 (Van de Bogart 1975). Clade VII is entirely comprised of species containing thick-walled, encrusted veil cells as well as pileal setules with capitate or swollen apex (*Coprinelluscurtus*, *Co.tenuis*). These differences between the clades found in our study and those in Nagy 2012 might therefore provide DNA phylogenic support for the morphologically defined section Domestici, but still leave the remaining sections in need of updating, clade VII being a separate *Curtus* clade.

In the present study, we demonstrated that low-altitude mountains and grasslands of Pakistan are rich in species of *Coprienllus*. The climatic conditions of these areas of the country are favourable for growth of coprinoid mushrooms. With the description of these four new species, the number of know species of *Coprinellus* from Pakistan increases to eight.

**Figure 4.**
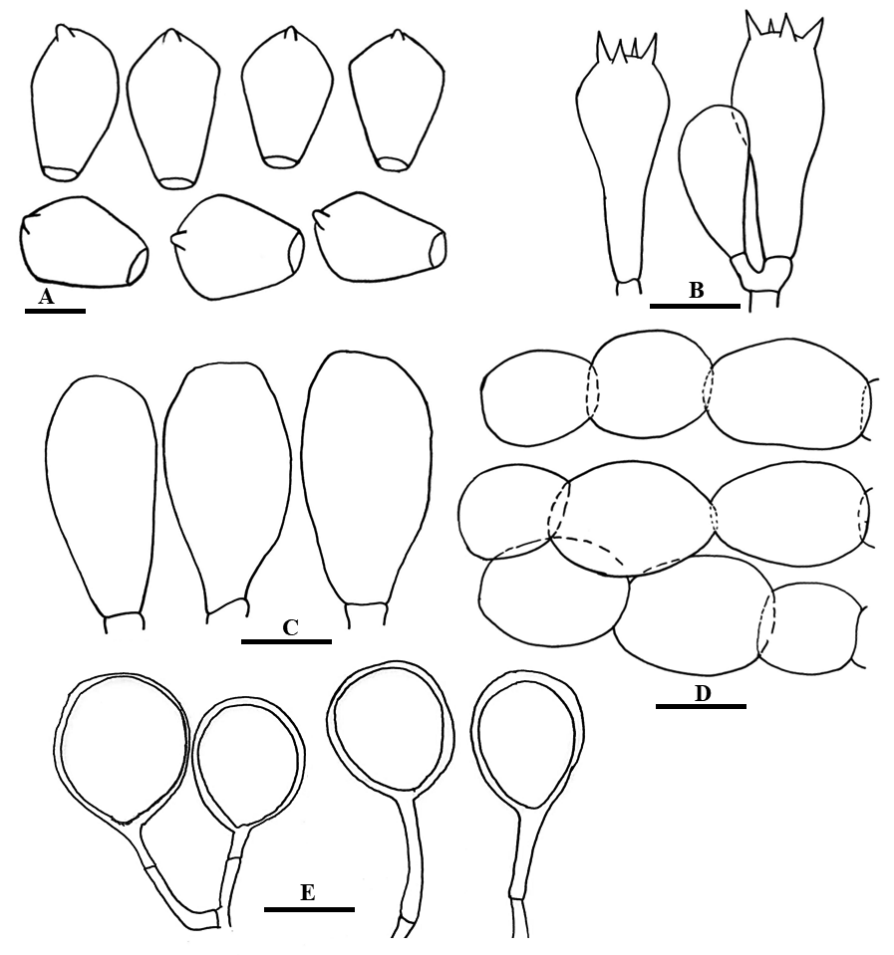
Line drawing of anatomical characters of *Coprinelluscampanulatus***A** Basidiospores **B** Basidia **C** Cheilocystidia **D** Pileipellis **E** Veil elements. Scale bars: 10 µm (**A**), 20 µm (**B–E**).

**Figure 5.**
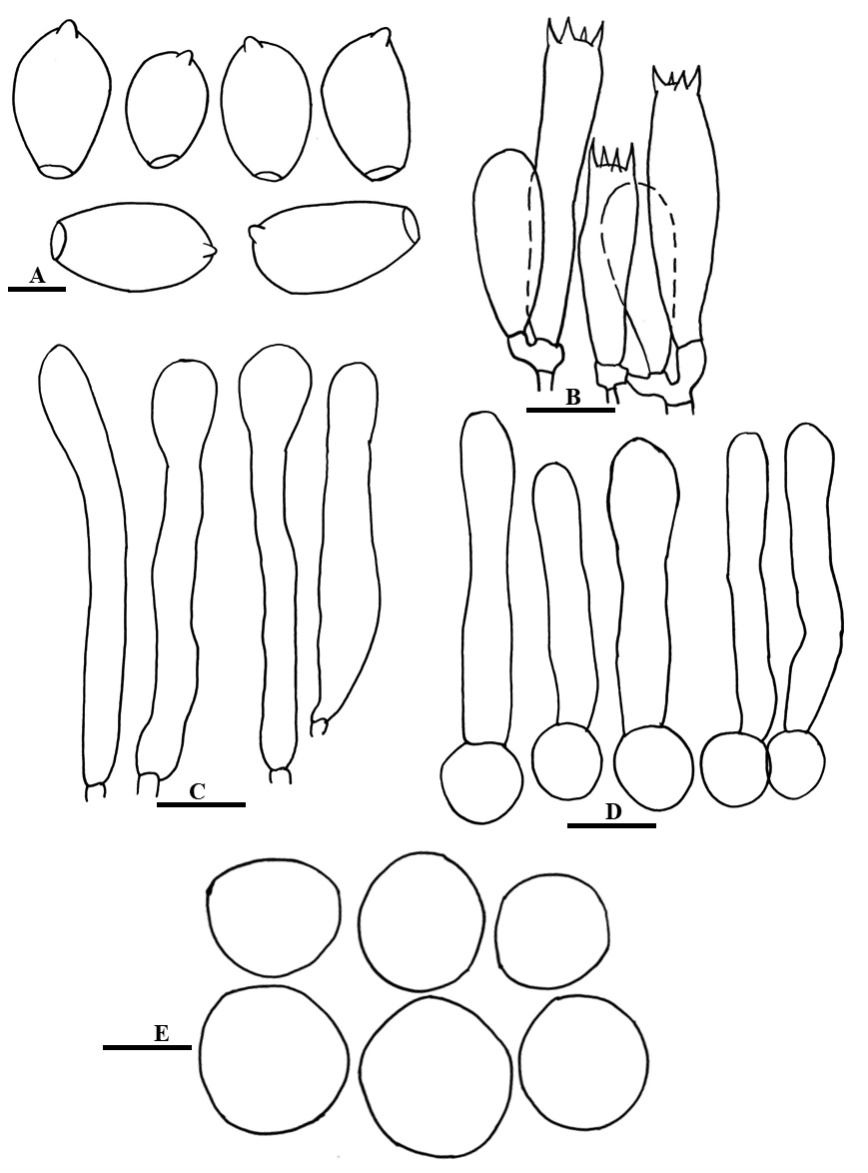
Line drawing of anatomical characters of *Coprinellusdisseminatus-similis***A** Basidiospores **B** Basidia **C** Cheilocystidia **D** Pileipellis with pileocystidia **E** Veil elements. Scale bars: 10 µm (**A**), 20 µm (**B–E**).

**Figure 6.**
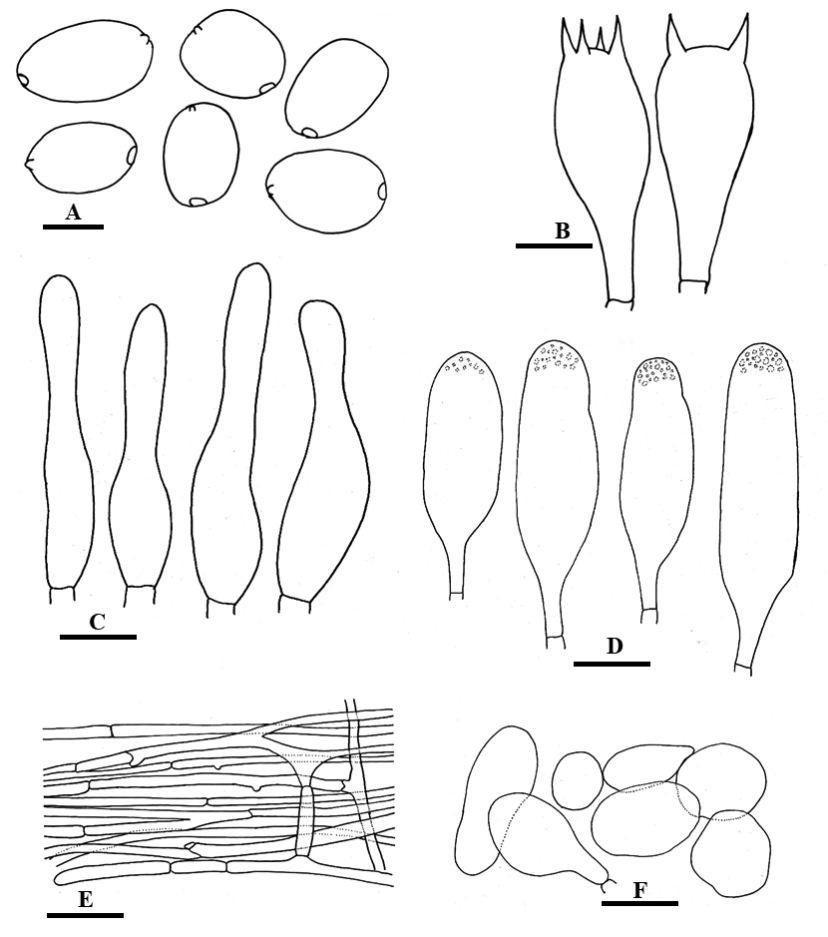
Line drawing of anatomical characters of *Coprinelluspakistanicus***A** Basidiospores **B** Basidia **C** Pileocystidia **D** Cheilocystidia **E** Pileal hyphae **F** Veil elements. Scale bars: 10 µm (**A**), 20 µm (**B–F**).

**Figure 7.**
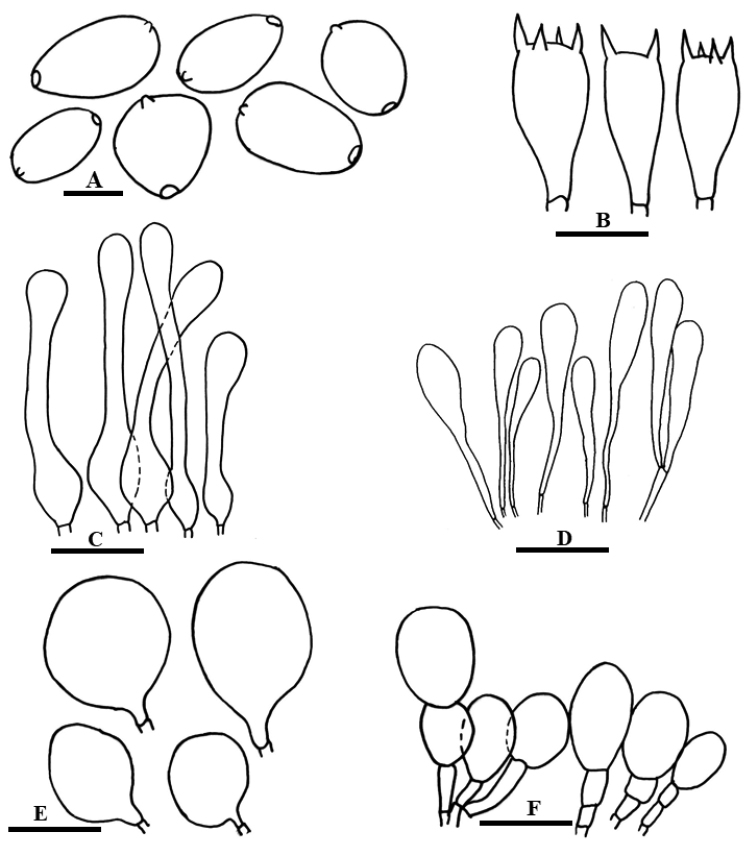
Anatomical features of *Coprinellustenuis***A** Basidiospores **B** Basidia **C** Pileocystidia **D** Caulocystidia **E** Cheilocystidia **F** Veil cells. Scale bars: 10 µm (**A**), 20 µm (**B–F**).

## Supplementary Material

XML Treatment for Coprinellus campanulatus

XML Treatment for Coprinellus disseminatus-similis

XML Treatment for Coprinellus pakistanicus

XML Treatment for Coprinellus tenuis

## References

[B1] Ahmad S (1980) A contribution to the Agaricales of Pakistan. Bulletin of Mycology 1: 35–90.

[B2] Drummond AJ Rambaut A (2007) BEAST: Bayesian evolutionary analysis by sampling trees. BMC Evolutionary Biology 7(1): 214. 10.1186/1471-2148-7-214 PMC2247476 17996036

[B3] Gernhard T (2008) The conditioned reconstructed process. Journal of Theoretical Biology 253(4): 769–778. 10.1016/j.jtbi.2008.04.005 18538793

[B4] Gomes ARP Wartchow F (2014) *Coprinellusarenicola*, a new species from Paraíba, Brazil. Sydowia 66: 249–256. 10.12905/0380.sydowia66(2)2014-0249

[B5] Hall TA (1999) BioEdit: a user-friendly biological sequence alignment editor and analysis program for Windows 95/98/NT. Nucleic Acids Symposium Series No. 41: Oxford University Press, 95–98. 10.1021/bk-1999-0734.ch008

[B6] Házi J Nagy GL Vágvölgyi C Papp T (2011) *Coprinellusradicellus*, a new species with northern distribution. Mycological Progress 10: 363–371. 10.1007/s11557-010-0709-y

[B7] Hussain S Afshan NS Ahmad H (2016) First record of *Parasolalilatincta* from Pakistan. Mycotaxon 131(2): 317–323. 10.5248/131.317

[B8] Hussain S Afshan NS Ahmad H Khalid AN Niazi AR (2017) *Parasolamalakandensis* (Psathyrellaceae; Basidiomycota) from Malakand, Pakistan. Mycoscience 58(2): 69–76. 10.1016/j.myc.2016.09.002

[B9] Hussain S Ahmad H Ullah S Afshan N Pfister DH Sher H Ali H Khalid AN (2018) The genus *Parasola* in Pakistan with the description of two new species. MycoKeys 30: 41–60. 10.3897/mycokeys.30.21430 PMC5904494 29681732

[B10] Keirle MR Hemmes DE Desjardin DE (2004) Agaricales of the Hawaiian Islands. 8. Agaricaceae: *Coprinus* and *Podaxis*; Psathyrellaceae: *Coprinopsis*, *Coprinellus* and *Parasola*. Fungal Diversity 15(3): 33–124.

[B11] Kirk PM Cannon PF Minter DW Stalpers JA (2008) Dictionary of the Fungi (10^th^ edn). CABI, Wallingford.

[B12] Larkin MA Blackshields G Brown NP Chenna R McGettigan PA McWilliam H Valentin F Wallace IM Wilm A Lopez R Thompson JD Gibson TJ Higgins DG (2007) ClustalW and ClustalX version 2.0. Bioinformatics 23(21): 2947–2948. 10.1093/bioinformatics/btm404 17846036

[B13] Matheny PB Curtis JM Hofstetter V Aime MC Moncalvo JM Ge ZW Yang ZL Slot JC Ammirati JF Baroni TJ Bougher NL (2006) Major clades of Agaricales: a multilocus phylogenetic overview. Mycologia 98(6): 982–995. 10.1080/15572536.2006.11832627 17486974

[B14] Munsell AH (1975) Munsell soil color charts. Baltimore, Munsell Color Inc., Baltimore.

[B15] Nagy GL (2008) Identification key to *Coprinus* species known from Europe. Clusiana 47: 31–44.

[B16] Nagy GL Vágvölgyi C Papp T (2010) Type studies and nomenclatural revisions in *Parasola* (Psathyrellaceae) and related taxa. Mycotaxon 112: 103–141. 10.5248/112.103

[B17] Nagy L (2011) An investigation of the phylogeny and evolutionary processes of deliquescent fruiting bodies in the mushroom family Psathyrellaceae (Agaricales). PhD Thesis, University of Szeged, Hungary.

[B18] Nagy LG Walther G Hazi J Vágvölgyi C Papp T (2011) Understanding the evolutionary processes of fungal fruiting bodies: correlated evolution and divergence times in the Psathyrellaceae. Systematic Biology 60(3): 303–317. 10.1093/sysbio/syr005 21368323

[B19] Nagy GL Hazi J Vágvölgyi C Papp T (2012) Phylogeny and species delimitation in the genus *Coprinellus* with special emphasis on the haired species. Mycologia 104: 254–275. 10.3852/11-149 21937727

[B20] Nagy GL Vágvölgyi C Papp T (2013) Morphological characterization of clades of the Psathyrellaceae (Agaricales) inferred from a multigene phylogeny. Mycological Progress 12: 505–517. 10.1007/s11557-012-0857-3

[B21] Örstadius L Ryberg M Larsson E (2015) Molecular phylogenetics and taxonomy in Psathyrellaceae (Agaricales) with focus on psathyrelloid species: introduction of three new genera and 18 new species. Mycological Progress 14(5): 25. 10.1007/s11557-015-1047-x

[B22] Orton PD Watling R (1979) Coprinaceae, Part 1: *Coprinus*. In: Henderson DM Orton PD Watling R (Ed.) British fungus flora Agarics and Boleti.Royal Botanic Garden, Edinburgh, 1–149.

[B23] Padamsee M Matheny PB Dentinger BT McLaughlin DJ (2008) The mushroom family *Psathyrellaceae*: evidence for large-scale phylogeny of the genus *Psathyrella*. Molecular Phylogenetics and Evolution 46(2): 415–429. 10.1016/j.ympev.2007.11.004 18248744

[B24] Prydiuk MP (2010) New records of dung-inhabiting Coprinus species in Ukraine I. Section Pseudocoprinus. Czech Mycology 62: 43–58.

[B25] Rambaut A Suchard MA Xie D Drummond AJ (2014) TRACER v 1.6. Computer program and documentation distributed by the authors. http://beast.bio.ed.ac.uk/Tracer [Accessed 18 Oct 2016]

[B26] Redhead SA Vilgalys R Moncalvo JM Johnson J Hopple JS (2001) *Coprinus* Pers.oon and the disposition of *Coprinus* species *sensu lato.* Taxon 50: 203–241. 10.2307/1224525

[B27] Schafer DJ (2010) Keys to sections of *Parasola*, *Coprinellus*, *Coprinopsis* and *Coprinus* in Britain. Field Mycology 11(2): 44–51. 10.1016/j.fldmyc.2010.04.006

[B28] Stamatakis A (2006) RAxML-VI-HPC: maximum likelihood-based phylogenetic analyses with thousands of taxa and mixed models. Bioinformatics 22(21): 2688–2690. 10.1093/bioinformatics/btl446 16928733

[B29] Tóth A Hausknecht A Krisai-Greilhuber I Papp T Vágvölgyi C Nagy LG (2013) Iteratively refined guide trees help improving alignment and phylogenetic inference in the mushroom family Bolbitiaceae PLoS One 8(2): e56143. 10.1371/journal.pone.0056143 PMC3572013 23418526

[B30] Uljé CB Bas C (1991) Studies in Coprinus II. Subsection Setulosi of section Pseudocoprinus. Persoonia 14(3): 275–339.

[B31] Uljé CB (2005) *Coprinus*. In: Noordeloos ME, Kuyper TW, Vellinga EC, eds. Flora Agaricina Neerlandica 6: 22–109.

[B32] Van de Bogart F (1975) The genus *Coprinus* in Washington and adjacent Western States. PhD Thesis, University of Washington, USA.

[B33] Vašutová M Antonin V Urban A (2008) Phylogenetic studies in Psathyrella focusing on section Pennatae and *Spadiceae* – new evidence for the paraphyly of the genus. Mycological Research 112(10): 1153–1164. 10.1016/j.mycres.2008.04.005 18786821

[B34] Walther G Garnica S Weiss M (2005) The systematic relevance of conidiogenesis modes in the gilled Agaricales. Mycological Research 109(5): 525–544. 10.1017/S0953756205002868 16018308

[B35] White TJ Bruns T Lee S Taylor J (1990) Amplification and direct sequencing of fungal ribosomal RNA genes for phylogenetics. In: Innis MA Gelfand DH Sninsky JJ White TJ (Eds) PCR Protocols: A Guide to Methods and Applications.Academic Press, San Diego, 315–322. 10.1016/B978-0-12-372180-8.50042-1

